# Supplementary material for: Dynamic influences on static measures of metacognition
Source: Nat Commun. 2022 Jul 21;13:4208. doi: 10.1038/s41467-022-31727-0 (PMC9301893; doi:10.1038/s41467-022-31727-0)
Supplement: Supplementary file 1 — Supplementary Information [file 41467_2022_31727_MOESM1_ESM.pdf]

Dynamic influences on static measures of metacognition

Supplementary Information

Desender et al.

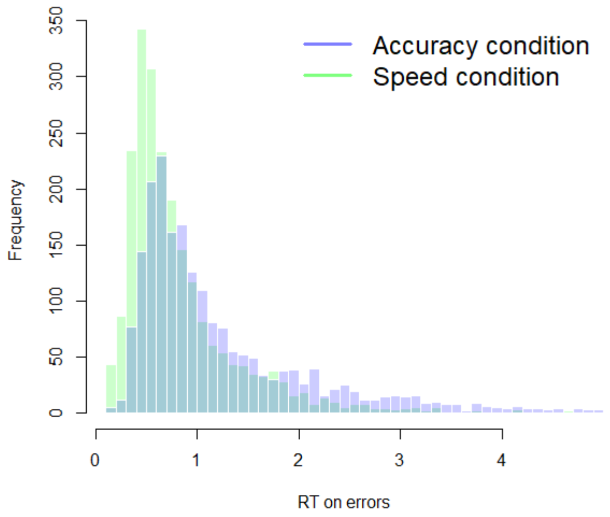

Supplementary Figure 1. Speed-accuracy tradeoff influences on error distributions. *The distribution of error RTs from Experiment 1, separately for the speed and the accuracy conditions reveals that our manipulation succesfully induced more fast errors in the speed condition and more slow errors in the accuracy condition. Source data are provided as a Source Data file.*

Supplementary Note 1: Replicating the effect of speed-accuracy tradeoffs on M-ratio

Here, we report the results of a replication study which was identical to Experiment 1 reported in the main manuscript, except that participants were instructed to provide confidence ratings on a continuous scale using the mouse (instead of with keyboard presses, as in Experiment 1). Although collecting confidence judgments with a continuous scale allows for a more fine-grained measurement of decision confidence, it comes at the cost that measurements of confidence RTs are less precise. Despite this methodological difference, results of this replication study were fully in line with those of Experiment 1.

In this replication study, we recruited 40 human participants (18 males, mean age = 19.82, between 18 and 30). Two participants were excluded because they required more than 10 practice blocks in one of the training blocks, and six participants were excluded because their choice accuracy was not different from chance level performance in at least one of both instruction conditions, as assessed by a chi square test. The final sample thus comprised thirty-two participants. The task was identical to Experiment 1, except for the following. Participants made a choice by pressing "E" or "T" with their left hand, corresponding to left and right choices, respectively. After the choice, and a blank screen of .5s, participants indicated their level of confidence using a continuous slider (see Supplementary Figure 2A). Consistent with the instructions, participants were faster in the speed condition than in the accuracy condition, M_{speed} = 738ms versus $M_{accuracy}$ = 992ms, $t(31) = 4.12$, $p < .001$, and numerically more accurate in the accuracy condition than in the speed condition, $M_{accurate}$ = 77.6% vs M_{speed} = 76.0%, $t(31) = 1.31$, $p = .20$. To test whether indeed errors in the speed condition were mostly "fast" errors and errors in the accuracy condition were mostly "slow" errors, despite a significant difference in overall error rate, we divided each participant's error RTs into three equal-sized bins (fast, medium or slow). We observed that in the fast bin there were more errors from the speed than from the accuracy condition (M_{speed} = 30.3 trials vs $M_{accuracy}$ = 14.0 trials, $t(31) = -5.73$, $p < .001$), whereas in the slow bin the reverse was true (M_{speed} = 17.1 trials vs $M_{accuracy}$ = 27.9 trials, $t(31) = 5.72$, $p < .001$). In the medium bin the number of trials did not differ between both instruction conditions (M_{speed} = 21.8 trials vs $M_{accuracy}$ = 22.7 trials, $p = .592$). Confidence RTs were not significantly different between the two instruction conditions, $p = .249$, suggesting participants selectively varied response caution for the motion coherence choice. The data further showed no significant between-participants correlations between median choice RTs and median confidence RTs in the accuracy condition, $r(30) = .325$, $p = .069$, nor in the speed condition, $r(34) = .08$, $p = .668$. Finally, participants were more confident in the accuracy condition than in the speed condition, $M_{accuracy}$ = 71 versus M_{speed} = 68, $t(31) = 3.04$, $p = .004$ (See Figure Supplementary Figure 2D).

Our bounded accumulation fitted the data well, as it captured the distributional properties of both reaction times and decision confidence (see Supplementary Figure 2C). As expected, decision boundaries were different between the two instruction conditions, M_{speed} = 1.47 versus $M_{accuracy}$ = 1.83, $t(31) = 3.95$, $p < .001$, suggesting that participants changed their decision boundaries as instructed. Also non-decision time tended to be a bit shorter in the speed condition compared to the accuracy condition, M_{speed} = 295ms versus $M_{accuracy}$ = 367ms, $t(31) = 2.33$, $p = .026$. Drift rates did not differ between both instruction conditions, $p = .207$. There was a small but significant difference between the two instruction conditions in the two additional parameters controlling the idiosyncratic mapping between $p(correct)$ and the confidence scale, reflecting that in the accuracy condition confidence judgments were slightly higher, $t(31) = 3.86$, $p < .001$, and less variable, $t(31) = 2.68$, $p = .012$, compared to the speed condition. Because confidence was expressed on a continuous scale, before fitting the meta-*d'* model (which requires categorical confidence judgments) confidence was divided into 4 equal-sized bins, separately for each participant. When trial counts were too imbalanced to create equal-sized bins, less bins were created. In line with the model simulations, our data showed that M-ratio was significantly affected by the speed-accuracy tradeoff instructions, M_{speed} = 0.66 versus $M_{accuracy}$ = 0.46, $t(31) = 2.95$, $p = .005$ (Supplementary Figure 2B). Consistent with the notion that

metacognitive accuracy should not be affected by differences in decision boundary, v-ratio did not differ between both instruction conditions, *p* = .575.

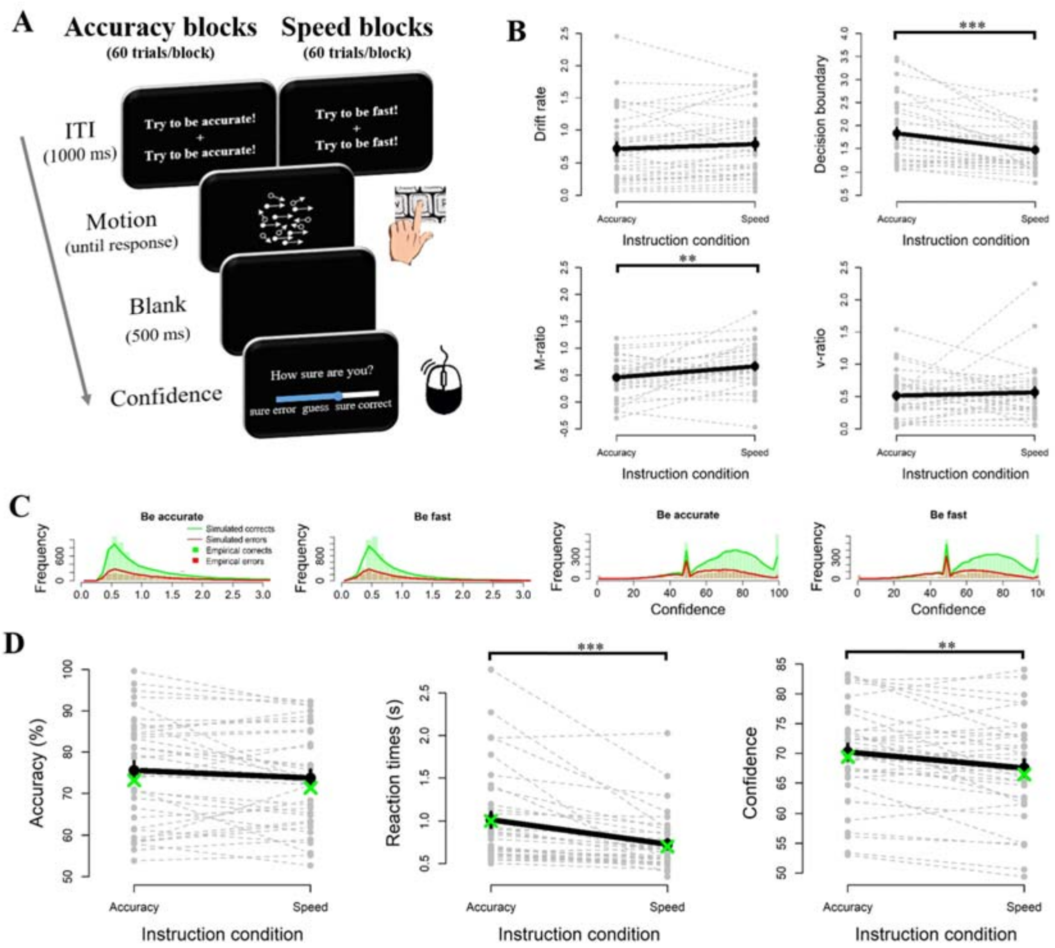

Supplementary Figure 2. The influence of speed-accuracy instructions on metacognitive accuracy. A. *Sequence of events in the experimental task. Participants (N=32) decided whether the majority of dots were moving left or right, by pressing "E" or "T" with their left hand. After a short blank, they then indicated their level of confidence on a continuous scale. Depending on the block, instructions during the ITI were either to focus on choice accuracy or to focus on speed.* ***B.*** *Fitted parameters of a drift diffusion model with additional post-decision accumulation. Fitted decision boundaries were lower in the speed vs accuracy condition, $t(31) = 3.95$, $p < .001$, whereas drift rates did not differ, $p = .207$. Critically, M-ratio was higher in the speed vs accuracy condition, $t(31) = 2.95$, $p = .005$, whereas v-ratio did not differ between both instruction conditions, $p = .575$.* ***C.*** *Distribution of reaction times and confidence for empirical data (bars) and model fits (lines), separately for corrects (green) and errors (red).* ***D****. Participants were faster, $t(31) = 4.12$, $p < .001$, and less confident, $t(31) = 3.04$, $p = .004$, when instructed to focus on speed rather than on accuracy, whereas accuracy itself did not significantly differ, $t(31) = 1.31$, $p = .20$. Note: grey lines show individual data points; black lines show averages; green dots show model fits; error bars reflect SEM; ***p<.001, **p<.01, *p<.05. Source data are provided as a Source Data file.*

Supplementary Note 2: Type II ROC as a function of post-decision drift rate

Here, we further analyzed the simulations reported in the main manuscript, by calculating area under type II ROC for each simulated agent, and plotted this against the simulated post-decisional drift rate (*v post*). Supplementary Figure 3A shows four representative type II ROC plots for different levels of post-decision drift rate. As can be seen in Supplementary Figure 3B, area under type II ROC is strongly related to post-decision drift rate, reaching an asymptote when post-decision drift rate is around ~ 1.5.

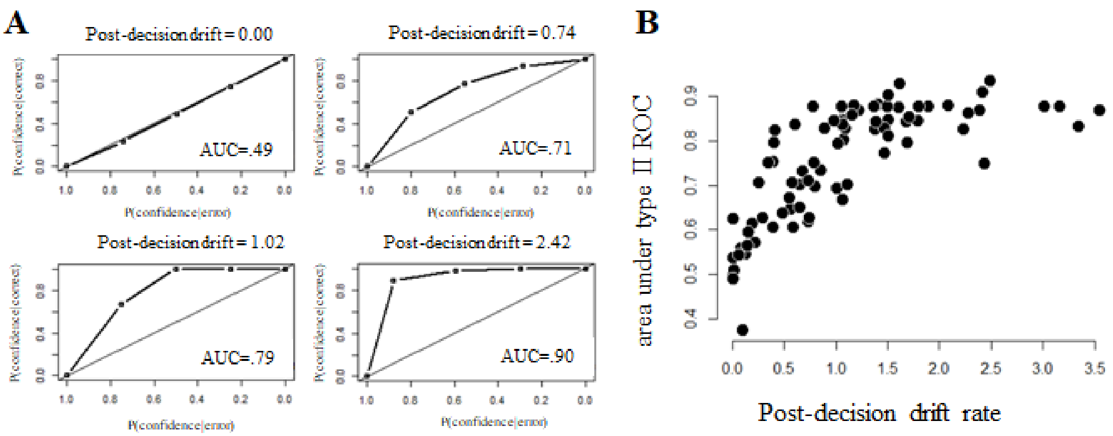

Supplementary Figure 3. *Four representative type II ROC plots for different levels of post-decision drift rate (A). The relation between type II ROC performance and post-decision drift rate (B).*

Supplementary Figure 3B suggests that when post-decision drift rate is zero, area under type II ROC is at chance level of .5. To further demonstrate this point, these simulations were performed again, but after fixing post-decision drift rate to zero. Indeed, these simulations showed that area under type II ROC does not significantly different from .5, M = .499, p = .807, *Bayes Factor* = 0.122. Thus, these additional simulations confirm that when post-decisional processing is zero, confidence judgments do not track the objective accuracy.
