# Supplementary material for: Dynamic influences on static measures of metacognition
Source: Nat Commun. 2022 Jul 21;13:4208. doi: 10.1038/s41467-022-31727-0 (PMC9301893; doi:10.1038/s41467-022-31727-0)
Supplement: Supplementary file 3 — Reporting Summary [file 41467_2022_31727_MOESM3_ESM.pdf]

nature research

Corresponding author(s): Desender, Kobe

Last updated by author(s): 2022/05/12

# Reporting Summary

Nature Research wishes to improve the reproducibility of the work that we publish. This form provides structure for consistency and transparency in reporting. For further information on Nature Research policies, see our Editorial Policies and the Editorial Policy Checklist.

## Statistics

For all statistical analyses, confirm that the following items are present in the figure legend, table legend, main text, or Methods section.

| n/a | Confirmed | |
|---|---|---|
| ☐ | ☒ | The exact sample size ($n$) for each experimental group/condition, given as a discrete number and unit of measurement |
| ☐ | ☒ | A statement on whether measurements were taken from distinct samples or whether the same sample was measured repeatedly |
| ☐ | ☒ | The statistical test(s) used AND whether they are one- or two-sided<br>*Only common tests should be described solely by name; describe more complex techniques in the Methods section.* |
| ☐ | ☒ | A description of all covariates tested |
| ☐ | ☒ | A description of any assumptions or corrections, such as tests of normality and adjustment for multiple comparisons |
| ☐ | ☒ | A full description of the statistical parameters including central tendency (e.g. means) or other basic estimates (e.g. regression coefficient) AND variation (e.g. standard deviation) or associated estimates of uncertainty (e.g. confidence intervals) |
| ☐ | ☒ | For null hypothesis testing, the test statistic (e.g. $F$, $t$, $r$) with confidence intervals, effect sizes, degrees of freedom and $P$ value noted<br>*Give P values as exact values whenever suitable.* |
| ☒ | ☐ | For Bayesian analysis, information on the choice of priors and Markov chain Monte Carlo settings |
| ☐ | ☒ | For hierarchical and complex designs, identification of the appropriate level for tests and full reporting of outcomes |
| ☐ | ☒ | Estimates of effect sizes (e.g. Cohen's $d$, Pearson's $r$), indicating how they were calculated |

*Our web collection on statistics for biologists contains articles on many of the points above.*

## Software and code

Policy information about availability of computer code

| | |
|---|---|
| Data collection | Data were collected using jsPsych, in which the experiment was programmed. |
| Data analysis | Data were analyzed using custom-written code in R (version 4.1.2), using the DEoptim library (version 2.2.6) for fitting purposes. All scripts have been deposited on osf and are freely accessible (https://github.com/kdesende/dynamic_influences_on_static_measures/). |

For manuscripts utilizing custom algorithms or software that are central to the research but not yet described in published literature, software must be made available to editors and reviewers. We strongly encourage code deposition in a community repository (e.g. GitHub). See the Nature Research guidelines for submitting code & software for further information.

## Data

Policy information about availability of data

All manuscripts must include a data availability statement. This statement should provide the following information, where applicable:

- Accession codes, unique identifiers, or web links for publicly available datasets
- A list of figures that have associated raw data
- A description of any restrictions on data availability

All raw data are deposited on osf and freely accessible (https://github.com/kdesende/dynamic_influences_on_static_measures/), or are part of the freely accessible confidence database (https://osf.io/s46pr/). In addition, Source Data are available for all figures.

# Field-specific reporting

Please select the one below that is the best fit for your research. If you are not sure, read the appropriate sections before making your selection.

- [ ] Life sciences
- [x] Behavioural & social sciences
- [ ] Ecological, evolutionary & environmental sciences

For a reference copy of the document with all sections, see nature.com/documents/nr-reporting-summary-flat.pdf

# Behavioural & social sciences study design

All studies must disclose on these points even when the disclosure is negative.

| | |
|---|---|
| Study description | Five datasets of quantitative experimental studies are reported in the current manuscript and one dataset in the supplementary materials. In all experiments, participants had to make perceptual decisions about random dot movements (Experiment 1 and 1S), about which of two circles contained more dots (Experiment 2A and 2C), about which array of gabor patches contained a pop-out element (Experiment 2B) or whether the average color of eight colored elements was red or blue (Experiment 2D). In all studies, participants made a binary response indicating their choice, followed by a rating of their confidence. |
| Research sample | Experiment 1: Forty-three healthy paticipants (16 males) took part in return for course credit (meange age = 19.2). Experiment 1S: Forty healthy participants (18 males) took part in return for course credit (mean age = 19.82, between 18 and 30). Experiment 2A was an existing dataset comprising 64 participants (50 female, mean age = 18.7, range 17-24), taking part in in return for course credit. Experiment 2B: ninety-nine healthy participants (10 males) took part in return for course credit (mean age = 18.5, between 18 and 21). Experiment 2C was an existing dataset comprising 204 participants (all female, age range 17-24). Experiment 2D was an existing dataset comprising 67 participants taking part in return for monetary compensation. Participants in Experiment 1, 1S and 2B were all 1st Bachelor students at KU Leuven. |
| Sampling strategy | For Experiment 1 and 1S, we used convenience sampling. No power analyses was performed, but we decided a prior to test 40 participants for Exp1S as this is common in the literature (e.g. Desender et al., 2021, Cognition), and aimed for the same number in Exp1. For Experiment 2B, we aimed for hundred participants in order to have sufficient power to detect small correlations (i.e., we had power of .8 to detect a correlation of .27 at an alpha level of .05). |
| Data collection | Because of COVID-19 data for Experiment 1, Experiment 1S, and Experiment 2B were collected online, using the jsPsysch library. Participants were blind to the study hypothesis. |
| Timing | Data for Experiment 1S were collected in April-May 2020, data for Experiment 1 were collected May 2021, data for Experiment 2B were collected February 2022. Data from the other experiments were reanalyses of previously published data, for which the timing of data collection is unknown. |
| Data exclusions | In experiment 1, one participant was excluded because they required more than 10 practice blocks in one of the training blocks and eight participants were excluded because their choice accuracy was not different from chance level performance in at least one of both instruction conditions, as assessed using a chi square test. Finally, two participants were excluded because they use the same confidence button in more than 95% of trials.<br>In experiment 1S, two participants were excluded because they required more than 10 practice blocks in one of the training blocks and six participants were excluded because their choice accuracy was not different from chance level performance in at least one of both instruction conditions, as assessed by a chi square test.<br>In experiment 2B, three participants were excluded because they used the same confidence button in more then 95% of trials. IN Experiment 2A,C-D no participants were excluded |
| Non-participation | No participants dropped out of the experiment or declined participation. |
| Randomization | Experiment 1 and 1S was fully within-subjects, so each participant took part in each condition (order counterbalanced across participants). Experiments 2A-D did not had any manipulations, so there was no allocation into experimental groups.. |

# Reporting for specific materials, systems and methods

We require information from authors about some types of materials, experimental systems and methods used in many studies. Here, indicate whether each material, system or method listed is relevant to your study. If you are not sure if a list item applies to your research, read the appropriate section before selecting a response.

## Materials & experimental systems

| n/a | Involved in the study |
|---|---|
| x | Antibodies |
| x | Eukaryotic cell lines |
| x | Palaeontology and archaeology |
| x | Animals and other organisms |
|  | x Human research participants |
| x | Clinical data |
| x | Dual use research of concern |

## Methods

| n/a | Involved in the study |
|---|---|
| x | ChIP-seq |
| x | Flow cytometry |
| x | MRI-based neuroimaging |

# Human research participants

Policy information about studies involving human research participants

| | |
|---|---|
| Population characteristics | See Above |
| Recruitment | Participants in Experiment 1, 1S, and 2B were recruited through the online participant management system SONA, contracted by KU Leuven. These participants were students of KU Leuven, who took part in return for course credit. We do not believe that this had an impact on the results. |
| Ethics oversight | Participants in Experiment 1, 1S, and 2B all provided informed consent before their participation. Ethical approval for these studies was granted by the local ethics committee at KU Leuven. The other experiments were reanalyses of previously published data. |

Note that full information on the approval of the study protocol must also be provided in the manuscript.
